# Pituitary Apoplexy Without Chiasmal Compression Presenting With Radiographic Oculomotor Nerve Compression in a Prolactinoma: A Case Report

**DOI:** 10.7759/cureus.111830

**Published:** 2026-06-30

**Authors:** Emily A Holz, Manal Imran, Nicole Reyes, Guillermo Izquierdo-Pretel

**Affiliations:** 1 Internal Medicine, Florida International University Herbert Wertheim College of Medicine, Miami, USA; 2 Hospital Medicine, Jackson Memorial Hospital, Miami, USA

**Keywords:** lactotroph pituitary adenoma, macroadenoma, oculomotor nerve (cn iii) palsy, pituitary apoplexy, transsphenoidal resection

## Abstract

Pituitary apoplexy is a rare but potentially life-threatening complication of pituitary macroadenomas that classically presents with acute headache, visual field deficits, and signs of optic chiasm compression. However, atypical neuro-ophthalmologic presentations without chiasmal involvement may delay recognition and treatment. This report highlights an unusual presentation of pituitary apoplexy associated with radiographic oculomotor nerve compression secondary to cavernous sinus extension in a patient with prolactinoma.

We report the case of a 29-year-old Hispanic woman who presented to the emergency department (ED) with acute right-sided headache, dizziness, and transient blurry vision after the reinitiation of cabergoline therapy for a previously diagnosed prolactinoma. The patient had a prolonged history of migraine-like headaches, amenorrhea, intermittent galactorrhea, and inconsistent adherence to dopamine agonist therapy due to medication intolerance. Magnetic resonance imaging (MRI) revealed a 1.5 cm right sellar macroadenoma with intralesional hemorrhage consistent with pituitary apoplexy. Imaging demonstrated extension into the right cavernous sinus with compression and lateral displacement of the oculomotor nerve, without contact or compression of the optic chiasm or optic nerves. Endocrine evaluation demonstrated hyperprolactinemia and secondary adrenal insufficiency. The patient underwent endoscopic transsphenoidal resection with subsequent clinical stabilization and normalization of prolactin levels. Pathology confirmed a lactotroph pituitary adenoma.

This case highlights an atypical presentation of pituitary apoplexy without classic chiasmal syndrome and emphasizes the importance of recognizing cavernous sinus cranial neuropathy as a potential presenting feature. Clinicians should maintain suspicion for pituitary apoplexy in patients with prolactinoma presenting with acute headache and cranial nerve (CN) abnormalities, even in the absence of visual field defects or optic pathway compression.

## Introduction

Pituitary adenomas are benign tumors of the pituitary gland, classified according to their size and whether they are hormone-secreting [1,2]. Macroadenomas are defined as having a diameter greater than or equal to 10 mm [1]. The estimated prevalence of pituitary macroadenomas in the general population is 0.2% [2]. They typically present with symptoms of mass effect from the tumor, including headache, visual field defects, or hypopituitarism [2,3]. Visual field defects often result from the compression of the optic chiasm, while hypopituitarism occurs because of either the compression of the pituitary stalk or functioning pituitary tissue [2]. Diagnosis is typically established with magnetic resonance imaging (MRI) of the sellar region along with endocrine evaluation to assess hormonal function [4]. Management depends on tumor size, hormonal activity, and the presence of compressive symptoms [4]. Dopamine agonists such as cabergoline are first-line therapy for prolactin-secreting tumors, while surgical resection via a transsphenoidal approach is often indicated for symptomatic macroadenomas or when medical therapy fails [4]. This report presents the case of a 29-year-old woman with a pituitary macroadenoma, seeking to address the following question: how can pituitary apoplexy be recognized when presenting with isolated oculomotor nerve compression without chiasmal symptoms in a patient with prolactinoma?

## Case presentation

A 29-year-old Hispanic woman presented to the emergency department (ED) with frontal right-sided headache, dizziness, and one day of transient blurry vision in the right eye. She has a six-year history of chronic “migraine-like” headaches. Approximately two years ago, the patient was diagnosed with a prolactinoma at a different hospital system. She reports three years of amenorrhea, which she initially attributed to a subdermal contraceptive implant; however, amenorrhea persisted after the removal of the implant. She reported one episode of galactorrhea approximately two years ago. This is what prompted her to seek medical care and ultimately led to her diagnosis with a pituitary macroadenoma. Following her diagnosis, the patient was started on cabergoline. However, the patient did not take cabergoline consistently. She would initiate and discontinue cabergoline numerous times, reporting that each time she began cabergoline, she would get a severe headache and lightheadedness. When the patient presented to the emergency department, she was two days post-resuming cabergoline and had one day of severe headache and blurry vision. A timeline of the patient’s presentation is seen in Figure 1.

**Figure 1:**
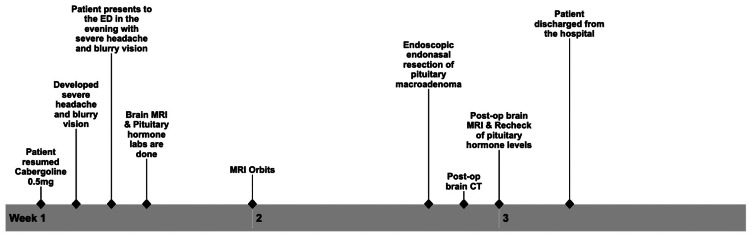
Timeline of Patient’s Clinical Course ED, emergency department; MRI, magnetic resonance imaging

On examination, vital signs were within normal limits. The patient was alert and oriented to person, place, and time. There was no facial asymmetry or weakness. Ophthalmologic examination revealed preserved visual acuity, intraocular pressure, and ocular motility, with right eye disc fullness and left eye pallor. There were no ptosis or pupillary changes; however, formal visual field testing was not performed.

Brain MRI performed on hospital day 1 revealed a 1.5 × 1.2 × 1.4 cm lesion within the right sella (Figure 2). The lesion was predominantly T2 hypointense with a T2 hyperintense component anteriorly. Findings were consistent with intralesional hemorrhage, concerning for apoplexy.

**Figure 2:**
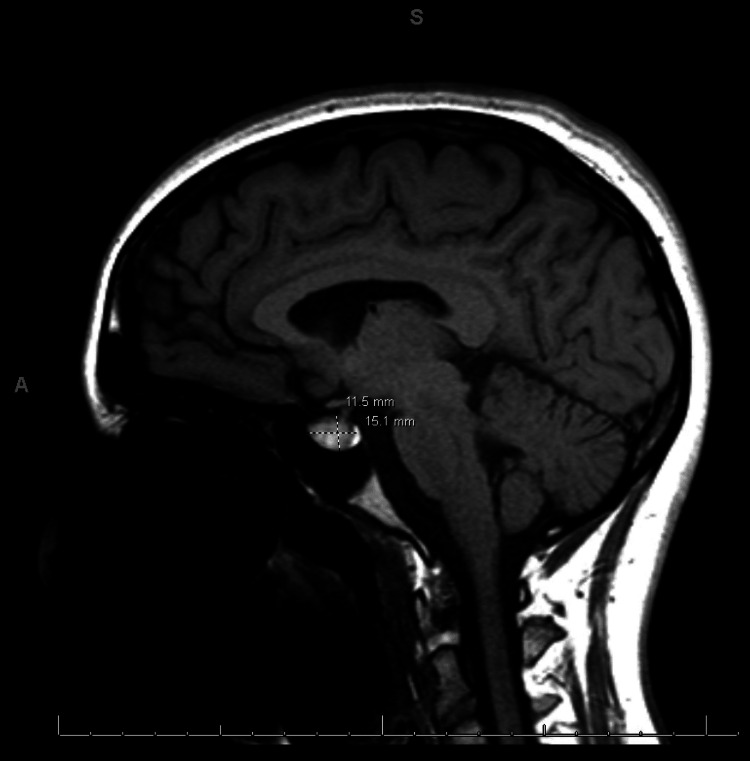
MRI of Prolactinoma MRI of the brain revealed a 1.5 × 1.2 × 1.4 cm lesion within the right aspect of the sella. There is heterogeneous T1 hyperintensity within the lesion, likely reflecting hemorrhagic components MRI: magnetic resonance imaging

Additionally, investigations revealed an elevated prolactin level, decreased adrenocorticotropic hormone (ACTH), decreased cortisol, decreased luteinizing hormone (LH), decreased follicle-stimulating hormone (FSH), normal thyroid-stimulating hormone (TSH), and normal free thyroxine (T4). Repeat free T4 was slightly low (Table 1).

**Table 1:** Patient’s Hormone Levels Upon Hospital Admission ACTH, adrenocorticotropic hormone; LH, luteinizing hormone; FSH, follicle-stimulating hormone; TSH, thyroid-stimulating hormone; T4, thyroxine

Laboratory test		Values	Reference range
Prolactin		257.5 ng/mL	3.0-25.0 ng/mL
ACTH		1.7 pg/mL	6-50 pg/mL
Cortisol		0.7 mcg/dL	4.0-22.0 mcg/dL
LH		<0.216 mIU/mL	0.5-16.9 mIU/mL
FSH		1.41 mIU/mL	1.5-17.7 mIU/mL
TSH		1.27 mIU/L	0.40-4.50 mIU/L
Free T4	Initial	0.87 ng/dL	0.8-1.8 ng/dL
	Repeat	0.77 ng/dL	

An MRI of the orbits was done on hospital day 3 (Figure 3). Imaging revealed that the pituitary lesion was overall stable in size. The lesion compressed and laterally displaced the right oculomotor nerve as it entered the cavernous sinus. There was no compression of or contact with the right optic nerve or optic chiasm. There were no signal abnormalities in either optic nerve, optic tract, or the optic chiasm. The overall impression was a pituitary macroadenoma with intralesional hemorrhage and extension into the right cavernous sinus with the compression of the oculomotor nerve and no compression of the optic nerve or optic chiasm. 

**Figure 3:**
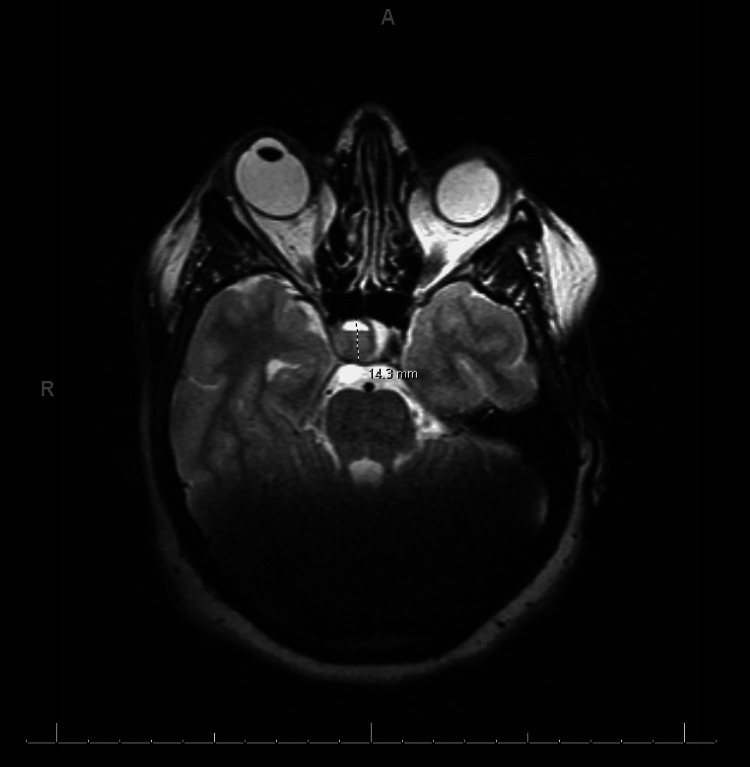
MRI of the Orbits MRI of the orbits reveals a stable pituitary lesion within the right sella. There is no mass effect on the optic chiasm MRI: magnetic resonance imaging

While pending surgery, the patient’s lacking hormones were replaced. She was initiated on hydrocortisone 50 mg IV every eight hours (q8h) and levothyroxine 50 mcg orally (po) daily. A decision was made for the transsphenoidal resection of the pituitary macroadenoma due to the patient’s presentation with visual field defects and concern for apoplexy.

The patient underwent an endoscopic endonasal transsphenoidal resection of a pituitary macroadenoma without complications. Two days after the procedure, her hydrocortisone dose was reduced to 25 mg IV q8h. A post-procedure MRI conducted on postoperative day 2 noted that residual pituitary tissue was present with the stalk mildly deviated to the left (Figure 4).

**Figure 4:**
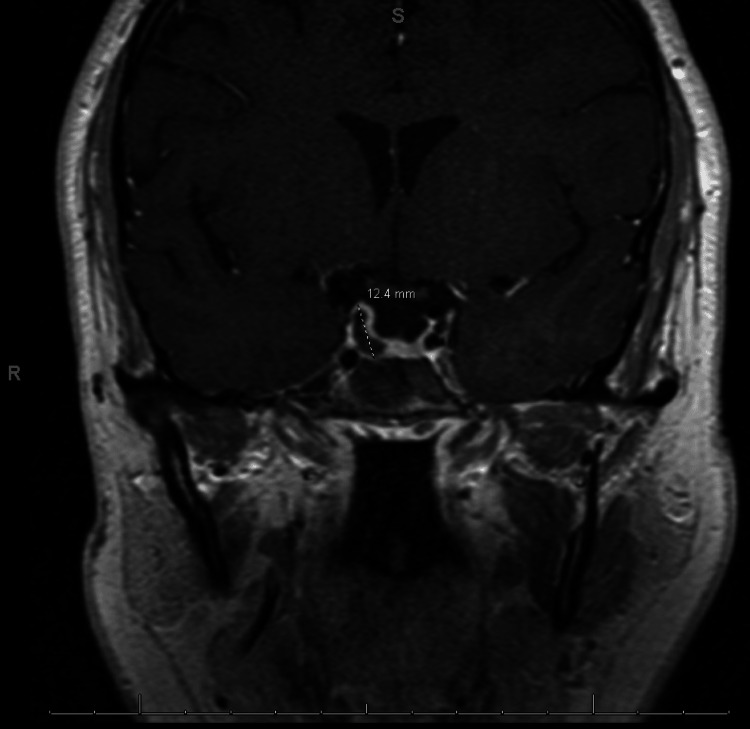
Postoperative Brain MRI There is a 1.2 × 1.0 cm T1/T2 dark cystic region along the right sella/cavernous sinus with linear peripheral enhancement, which likely reflects postoperative change MRI: magnetic resonance imaging

Additionally, her prolactin and free T4 were reassessed following the removal of the macroadenoma (Table 2). Postoperative prolactin and free T4 were within normal limits. The patient was discharged home on hydrocortisone 5 mg and levothyroxine 25 mcg.

**Table 2:** Postoperative Hormone Levels T4: thyroxine

Laboratory test	Values	Reference range
Prolactin	3.2 ng/mL	3.0-25.0 ng/mL
Free T4	1.5 ng/dL	0.8-1.8 ng/dL

Following discharge, pathology reports were obtained. Pathology identified a lactotroph pituitary adenoma, consistent with a prolactinoma. The specimen stained positive for prolactin and PIT-1, and stains for other pituitary hormones, including ACTH, FSH, and TSH, were negative, confirming a prolactin-secreting macroadenoma.

## Discussion

About half of patients who present clinically with pituitary tumors have macroadenomas [1]. In a five-year follow-up of patients with nonfunctioning macroadenomas, the growth of the macroadenoma will be seen in approximately 50% of patients, and symptomatic pituitary apoplexy will develop in approximately 10% of patients [2]. Pituitary apoplexy is a clinical syndrome associated with either infarction or hemorrhage of a preexisting pituitary macroadenoma, characterized by acute-onset headache that may be accompanied by visual disturbance or cranial nerve (CN) palsy [5,6]. While often spontaneous, reported precipitants of pituitary apoplexy include head trauma, radiotherapy, coagulation disorders, and the initiation or withdrawal of dopamine agonist therapy [5].

Pituitary macroadenomas classically cause bitemporal visual field defects due to the compression of the optic nerve [2]. However, this patient did not have the compression of the optic nerve; rather, she presented with compression of the oculomotor nerve. While apoplexy may be classically thought of as presenting with chiasmal syndrome, it is important to note that apoplexy can present without chiasmal syndrome. For patients with pituitary adenomas, the compression of the optic nerve is seen in 30%-70% of cases [7], while oculomotor nerve palsies are seen in 1%-14% of cases [8].

The Pituitary Society’s consensus statement regarding prolactin-secreting pituitary adenomas notes that dopamine agonist therapy is highly effective, with cabergoline being the preferred dopamine agonist [9]. Recommendations for the surgical resection of pituitary adenomas are dependent on the size of the adenoma and the likelihood of cavernous sinus invasion, which is assessed via the Knosp classification [9]. However, for patients with rapidly progressive vision loss due to either mass effect or apoplexy, surgery may be recommended as first-line treatment over medical treatment with dopamine receptor agonists regardless of Knosp grade [9]. For patients with visual field defects, transsphenoidal resection is the treatment of choice as it is the only treatment modality that immediately decompresses visual pathways [2,9]. The Pituitary Society’s guidelines also note that surgery may be indicated for patients with intolerance or resistance to dopamine receptor agonist therapy; however, this is classified as a weaker recommendation [9].

Important limitations of this report must be noted. First, the absence of formal visual field testing should be taken into consideration. Formal visual field testing may assist with the early detection of compression, assessment of severity, and monitoring of medical treatment success. Additionally, this report presents a single case; thus, the information presented in this report lacks generalizability and cannot establish causality.

However, the takeaway points from this case are important for the care of future patients. Recognition and early evaluation are critical, with regular assessments of pituitary functioning being needed in patients managed with a conservative medical therapy approach. Additionally, while macroadenomas are typically thought of as affecting cranial nerve II, it is important for physicians to be cognizant of atypical CN involvement.

## Conclusions

This case highlights a patient presenting with a pituitary macroadenoma with signs of apoplexy following the repeated initiation and withdrawal of dopamine agonist therapy. This patient’s presentation was atypical in that she presented with pituitary apoplexy without classic chiasmal syndrome. This case emphasizes the importance of clinicians maintaining suspicion for pituitary apoplexy even without chiasmal signs when cranial neuropathy or invasion of the cavernous sinus is present.
